# Integrating single-cell transcriptomics with cellular phenotypes: cell morphology, Ca^2+^ imaging and electrophysiology

**DOI:** 10.1007/s12551-023-01174-2

**Published:** 2023-12-18

**Authors:** Joan Camunas-Soler

**Affiliations:** 1 Department of Medical Biochemistry and Cell Biology, Institute of Biomedicine, University of Gothenburg, 405 30 Gothenburg, Sweden; 2 Wallenberg Centre for Molecular and Translational Medicine, Sahlgrenska Academy, University of Gothenburg, 405 30 Gothenburg, Sweden

**Keywords:** Single-cell, Morphology, Phenotypes, Patch-seq, Imaging, Calcium, Transcriptomics, Cell-type, Excitability, Function

## Abstract

I review recent technological advancements in coupling single-cell transcriptomics with cellular phenotypes including morphology, calcium signaling, and electrophysiology. Single-cell RNA sequencing (scRNAseq) has revolutionized cell type classifications by capturing the transcriptional diversity of cells. A new wave of methods to integrate scRNAseq and biophysical measurements is facilitating the linkage of transcriptomic data to cellular function, which provides physiological insight into cellular states. I briefly discuss critical factors of these phenotypical characterizations such as timescales, information content, and analytical tools. Dedicated sections focus on the integration with cell morphology, calcium imaging, and electrophysiology (patch-seq), emphasizing their complementary roles. I discuss their application in elucidating cellular states, refining cell type classifications, and uncovering functional differences in cell subtypes. To illustrate the practical applications and benefits of these methods, I highlight their use in tissues with excitable cell-types such as the brain, pancreatic islets, and the retina. The potential of combining functional phenotyping with spatial transcriptomics for a detailed mapping of cell phenotypes in situ is explored. Finally, I discuss open questions and future perspectives, emphasizing the need for a shift towards broader accessibility through increased throughput.

## Introduction

**Fig. 1:**
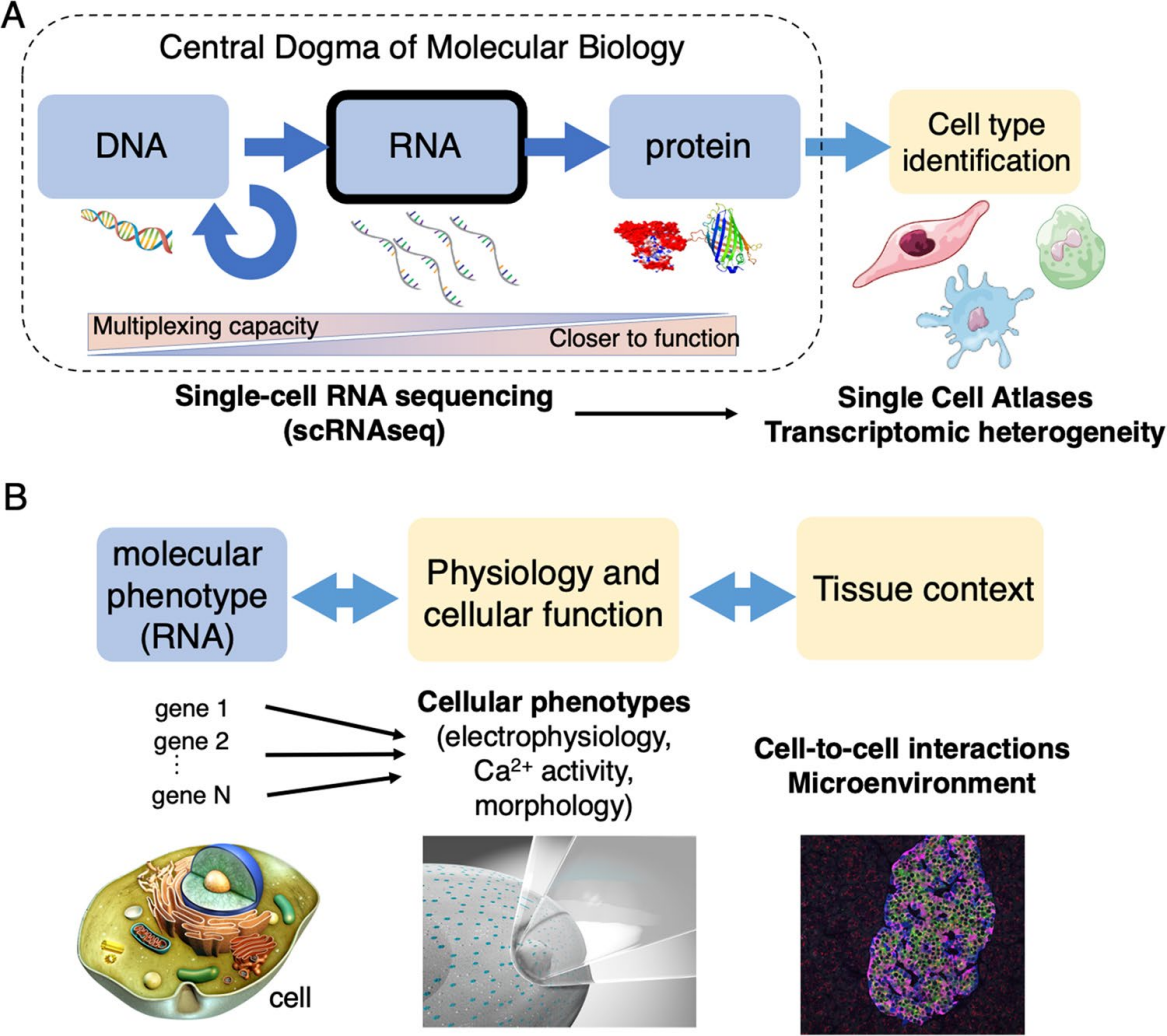
Coupling single-cell RNA sequencing (scRNAseq) to biophysical measurements of cellular physiology. **(A**) Technologies for molecular phenotyping of cells in the context of the central dogma of molecular biology. The advent of next-generation sequencing enabled high sensitivity multiplexed measurements of DNA and RNA in biological samples at single-cell resolution. Overall, scRNAseq provides a trade-off between (i) the ability to perform a precise molecular characterization of many cells and (ii) being a proxy of protein expression (which is closer to molecular function).This has been mostly used to construct reference atlases of cell types across organisms and to identify transcriptomic variability within cell types. **(B)** Interpretation of these molecular characterizations is challenging. Combination of direct measurements of cellular responses (as a proxy of function) and scRNAseq enables the direct identification of candidate genes and pathways with functional roles using unbiased transcriptome-wide analysis. Some of these methods allow to perform these measurements in situ, where cell-to-cell interactions are preserved

Single-cell RNA sequencing (scRNAseq) is a unique tool to perform cell type classifications based on their transcriptional profile (Manno et al. 2016; The Tabula Muris Consortium 2018; Villani et al. 2017; S. R. Quake 2021; Regev et al. 2017). This technique provides increasingly accurate cell type classifications in diverse organs, by measuring the transcriptome of individual cells (Fig. 1A). It has transformed our understanding of cellular diversity and heterogeneity, enabling the identification of rare and previously unknown cell types and states. However, challenges persist in directly attributing physiological properties in a cell to its measured transcriptome and in contextualizing this information within the tissue structure and microenvironment (Mayr et al. 2019; Kravets and Benninger 2020). For instance, it remains unclear whether distinctions based on molecular methods such as scRNAseq align with those obtained using morphological profiling and physiological and functional assays.

In this review, I discuss methods that aim to bridge this gap by integrating single-cell transcriptomics with biophysical measurements of cellular function in the same cell (Fig. 1B). An essential application of these multimodal technologies is the ability to identify physiologically relevant cellular states. They can also refine cell type classifications and identify cellular subtypes that are overlooked by individual modalities—hence contributing to a more comprehensive understanding of tissue and cell heterogeneity.

## Enhancing scRNAseq with cellular phenotypes

A critical aspect when designing a multimodal single-cell experiment is the extent to which information of ‘cell state’ can be extracted with each new data modality. Three factors to guide this selection are (i) the timescale of fluctuations in gene expression in relation to the measured phenotypes, (ii) the shared information content between modalities, and (iii) the analytical tools required to retrieve this information. Table 1 provides an overview of phenotypical characterizations that have been combined with scRNAseq, along with their respective strengths and limitations. Further discussion on cell morphology, calcium (Ca^2+^) imaging, and electrophysiology is presented in the following sections.

**Table 1:** Overview of phenotyping techniques combined with scRNAseq or spatial transcriptomics

Phenotypical characterization	Methods	Tissues / cell types	Time-resolution of cell activity	Throughput	Co-registration in same cell or tissue slice possibe	Barriers to entry	Other considerations
Morphology	Optical imaging	Most tissues	Low	Low/Medium	Yes	Low	Most accesible method
	EM ultrastructure				No	High	Destructive method ^b^ Specialized equipment ^c^
Chemical composition	Raman Spectroscopy	Most tissues	Low	Low/Medium	Yes	High	Specialized equipment ^c^
	MALDI-MSI				No	High	Destructive method ^b^
Ca2 + imaging and fluoresence	Ca^2+^ dyes	Excitable cell types or cells with Ca^2+^ signaling ^a^	Medium/High	Medium/High (with FACS)	Yes	Low	Lower specificity than other methods
	Voltage or TRAP sensors				Yes	Medium	Genetically encoded
Electrophysiological measurement	patch-seq	Excitable cell types (e.g. neurons, cardiomyocites, islets, retina)	High	Low	Yes	High	Specialized equipment

^a^Fluorophores targeting other measurements of cell function could be used in other cell types

^b^Requires a contiguous tissue slice (or equivalent cell) for transcriptomics

^c^Usually accesible in core facilities

### Timescales

Various techniques are employed to track phenotypical changes in cells across different timescales. Morphodynamics, for instance, monitors changes occurring over minutes to days (Copperman et al. 2023). Ca^2+^ imaging measures oscillations from tens of milliseconds to minutes, and electrophysiology records cellular activity down to the millisecond, such as action potential firing (Clapham 2007; Kulkarni and Miller 2017). Other methods such as electron microscopy and mass spectroscopy can capture a high-resolution view of organelle morphology and chemical species respectively. However, these are destructive methods and less suited for capturing cell dynamics (Table 1).

Most of these biophysical properties are predominantly shaped by the cell’s molecular constituents—metabolites, mRNA, proteins—each characterized by different turnover speeds. The mRNA pool in a cell is determined by its transcription, splicing rates (minutes) and nuclease degradation (hours) (Milo and Phillips 2015). Positioned between faster-turning metabolites and longer-lived proteins, mRNA serves as a versatile tool for capturing molecular snapshots of cellular ‘states’. Additionally, by estimating the abundance of spliced and unspliced mRNAs, termed RNA velocity, it becomes possible to capture longer dynamical processes or transient cellular states (Manno et al. 2018). While the focus of this review centers on scRNAseq, complementary techniques such as scATAC-seq could provide valuable insight into biophysical properties and phenotypes with slower fluctuations.

### Information content

Quantifying the relationship between mRNA abundance and emerging cellular phenotypes is technically challenging and remains relatively unexplored. In a study conducted on human cell lines, various features of global cell state—such as cell size, cell cycle state, and Ca^2+^ signaling—were measured alongside single-cell gene expression (Foreman and Wollman 2020). A linear model incorporating 13 of these features could explain between 15 and 85% of the measured variance in gene expression, with a median explanation of 62%. Notably, cell size exhibited the highest explanatory power, followed by Ca^2+^ signaling and cell cycle state. Although some Ca^2+^ features had a modest effect on the explained variance, most genes exhibited significant correlations with at least one Ca^2+^ feature, suggesting non-random associations (Foreman and Wollman 2020).

In a subsequent study, information theory was employed to reveal that, conversely, 60% of Ca^2+^ signaling dynamics could be explained by 83 genes, each contributing up to 17% of the signal. This highlights substantial redundancy within gene expression networks, hinting that cell state may be effectively represented by a few latent dimensions (Maltz and Wollman 2022). While cell lines may display considerable fluctuations in phenotype and RNA abundance, they are isogenic populations representing generally homogenous groups (Emert et al. 2021). Consequently, exploring transcriptome-wide measurements alongside functional phenotyping in primary cells may shed new light into this question.

### Analysis

Integrating scRNA-seq with functional phenotypes presents challenges due to the sparsity and intrinsic noise of the data. A prevalent strategy is employing unsupervised methods such as principal component analysis, followed by hierarchical clustering or low-dimensional embeddings (e.g. UMAP) to segregate data into distinct phenotypical clusters. Subsequent steps involve using differential expression analysis and statistical tests to identify transcripts enriched in each cluster. In some cases, the reverse approach also proves fruitful. For instance, exploring functional differences between cells selected on their expression of lineage transcription factors led to the discovery of dysfunctional cell subtypes in diabetes (Dai et al. 2022). Sparse regression (similar to PCA) has also been proposed for obtaining interpretable visualizations of paired transcriptomic and electrophysiological data (Kobak et al. 2021).

Correlative analysis between functional phenotypes and gene expression data is another valuable tool (Camunas-Soler et al. 2020). The large number of genes can complicate obtaining meaningful results due to the numerous hypotheses tested. To address this, selecting highly variable genes or employing biology-based gene curation can refine the initial gene pool. Non-parametric tests like Spearman correlation help mitigate issues related to outlier genes. Leveraging information theory tools, such as mutual information, can identify features with non-monotonic trends (Maltz and Wollman 2022). Typically, these approaches yield a subset of target genes that can be validated in independent experiments or used for modeling.

Training of machine learning models is another useful tool to identify features with predictive power across data modalities (Wang et al. 2023a). Linear models with intrinsic feature selection (such as Lasso) are a useful starting point. More complex non-linear models such as random forests or neural nets can refine modeling once an initial set of genes is determined. A challenge usually arises from the extensive gene space (more features than samples) which is even larger when combined with the functional phenotype data, making overfitting likely. This challenge can be addressed through cross-validation methods and holding an independent set. Lastly, network-based analysis can be used to leverage correlation structures between gene modules to enhance the predictive power of models (Camunas-Soler et al. 2020).

## Cell morphology

Cell morphology is a fundamental feature to distinguish cell types and is the basis for modern neuronal taxonomy (S. Ramon y Cajal, L. Azoulay 1955). Morphological properties of cells such as size, shape, granularity, and density of subcellular compartments dynamically respond to external perturbations (Chen et al. 1997). Hence, cell morphology has been extensively used in biomedical applications, including cell type classification, compound toxicity screening, and assessing metastatic capacity and responses to drug treatments (S. Ramon y Cajal, L. Azoulay 1955; Loo et al. 2007; Zink et al. 2004; Minn et al. 2005). Accessible through techniques like bright-field microscopy, morphological characterization does not require highly specialized equipment. However, in specific fields such as neuroscience, morphological analysis often involves patch-clamp electrophysiology, which is discussed in a separate section below.

A common method for conducting morphological analysis is high-content image-based screening or morphological profiling. Morphological profiling uses automated digital microscopy to quantify thousands of morphological features across multiple cells. This approach has been valuable in characterizing genes and compounds in both genetic and chemical perturbation assays (Loo et al. 2007; Bray et al. 2016; Liberali et al. 2014; Yin et al. 2013; Laufer et al. 2013; Caicedo et al. 2017). Integration with independent gene expression screens, through bulk RNA sequencing, enables the creation of extensive compendia of perturbation experiments and systematic functional studies (Nassiri and McCall 2018; Subramanian et al. 2017; Wawer et al. 2014). However, until recently, the combination of morphological imaging with scRNAseq has not been systematically used due to the complexity to co-register both measurements. Despite technical challenges, simultaneous morphological and molecular analysis within the same cell can illuminate fundamental mechanisms of cell function and homeostasis. An early scRNAseq study, for example, uncovered a scaling factor between global transcriptional activity and cell volume in mammalian cells using paired imaging and transcriptomics (Padovan-Merhar et al. 2015). Since then, the relationship between cell morphology and gene expression has been explored in greater detail, incorporating more general morphological features of cell state (Foreman and Wollman 2020).

### Methodological aspects

In a typical workflow, cells are initially imaged using bright-field microscopy, and subsequently each cell is independently collected for scRNAseq. However, this requirement for individual cell isolation hinders throughput and scalability. Some approaches for cell picking and processing include micropipette aspiration methods (Camunas-Soler et al. 2020; Cadwell et al. 2016; Tang et al. 2009), capture microdissection (Espina et al. 2006), microwells (Gong et al. 2010; Yuan et al. 2018), optofluidic transport (Berkeley Lights) (Jorgolli et al. 2019), hydrogel-well embedding (Lee et al. 2022), magnetic rafts (Gach et al. 2011), classic microfluidic valve-based system (Marcus et al. 2006; Wu et al. 2014), and image-based single-cell isolation (Shomroni et al. 2022). A comprehensive review of these approaches can be found in Fung et al. (2020). The choice of the optimal system for cell picking depends on the microscopy setup and the cell type under investigation. Micropipette aspiration methods are well-suited to detach adherent cells from microplate surfaces, while nanowells and microfluidic chambers excel at confining and processing free-floating cells in suspension. Several semi-automated cell-picking systems, inspired by earlier cell colony pickers, have achieved commercial success (e.g. CellCellector, Cellenion) (Shomroni et al. 2022; Nelep and Eberhardt 2018).

An elegant alternative to pairwise measurements in the same cell, is the coupling of droplet-based single-cell transcriptomics to image-based screens of organoids. In this approach organoids are classified based on their morphological profile (morphotype) and subsequently dissociated to perform scRNAseq in cells from each morphotype (Jain et al. 2023). Applying this methodology, Liberali and colleagues screened thousands of intestinal organoids against 301 compounds to identify 15 characteristic organoid phenotypes by imaging (Lukonin et al. 2020). In this way, they found a compound that induces a fetal-like regenerative state in enterocytes and measure its transcriptomic profile. A limitation of this approach is that it cannot establish direct correlations between morphology and gene expression in each cell but rather only at the population level. However, it is a powerful approach to identify transcripts enriched in rare cell populations present in morphologically defined organoids.

### Towards high-throughput methods

An exciting frontier in this field is the development of high-throughput approaches for multidimensional morphological analyses in real-time, coupled with single-cell sorting. Recently, deep-learning-assisted image-activated cell sorting demonstrated real-time sorting of algal and blood cell populations based on intracellular proteins (Nitta et al. 2018). This method, akin to an imaging version of FACS, relies on fast software-hardware infrastructure to minimize latency time for cell sorting (i.e. the time needed to analyze each cell image and deflect the cytometry stream to sort the cell) (Isozaki et al. 2020). This enables the sorting of cells based on morphological and biological characteristics such as size, shape, subcellular structures, blebbing or pigmentation. An in-depth review on image-based live-cell sorting can be found in LaBelle et al. (2021). Image-activate cell sorting extends beyond single-cell studies, and can be used to track cell-to-cell interactions. For example, it has served as a platform for screening T cell activation and binding to target tumor antigens (Segaliny et al. 2018). A similar method has recently been combined with the analysis of surface molecular markers using cytometry (Mavropoulos et al. 2023), and in principle, scRNAseq of pre-sorted morphologically defined cell subpopulations is also possible (Salek et al. 2022). To our knowledge, live-cell image-based cell sorting has not been coupled to single-cell sequencing on a cell-by-cell-basis yet.

### In silico integration

Methods aiming to integrate single-cell transcriptomics and morphology in silico seek to overcome the technical challenge of experimentally coupling both measurements. An illustrative example is the augmentation of imaging cell cytometry datasets using scRNAseq data (Chlis et al. 2020). In silico integrations are not multimodal experiments, as both data modalities are measured in independent sets of cells. In some cases, these cells could have been collected on different days or from different labs. Nevertheless, they are useful to characterize well-defined cell types or subtypes.

A crucial step in in silico integrations is the approach used to overlay both datasets together. Typically, this is achieved by co-registering both datasets using a common subset of cell surface markers. These markers must be available in both imaging and transcriptomic datasets, and are used to align cells with their nearest neighbors in marker gene expression (Chlis et al. 2020). The limited number of shared marker genes across both datasets poses a challenge for these bioinformatic integrations. For instance, in Chlis et al. (2020) only two cell surface marker genes could be used (CD34 and FcgR). The development of large panels of marker genes that can be registered in both data modalities would improve the applicability of these methods.

### Spatial transcriptomics integration with morphology and chemical analysis

The combination of spatial transcriptomics and histological imaging is enabling a new generation of approaches to study the connection between transcriptome and cell morphology in situ (Ståhl et al. 2016). Spatial transcriptomics can circumvent some of the outlined difficulties to isolate single cells after morphological characterization. It also provides additional information such as orientation, cellular interactions, and tissue microenvironment. Some challenges and limitations of spatial transcriptomics are as follows: (i) achieving single-cell resolution and screening large tissue sections simultaneously, (ii) cell segmentation, (iii) slice preparation and conservation, (iv) the trade-off between multiplexing capacity and sensitivity, and (v) its higher price point when using commercial options. From an experimental perspective, the choice of spatial transcriptomics technology is critical and will determine if the morphological analysis can be performed in the same slice or in an adjacent slice. A comparison of current methods can be found in Vandereyken et al. (2023), and new technologies with increased sensitivity and resolution are emerging fast.

Most spatial transcriptomics methods require thin slice preparations (10 μm) to access the RNA content of individual cell layers. This means that co-registration in consecutive tissue slices provides information of the same morphological structures and cellular neighborhood. However, when an adjacent slice needs to be used to collect both data modalities, data overlaying becomes complex. Artifacts related to tissue shearing, damage, and anisotropic deformation during thin slice preparation complicate data integration. Large structural features can be used for alignment, but the lack of a ground truth reference in short length scales is a common challenge. Methods borrowed from computed tomography, such as diffeomorphic metric mapping, are being used to align slices in the presence of tissue tearing (Clifton et al. 2023). Other developments in the analysis of these datasets include the use of deep-learning architectures. These architectures aim to learn low dimensional joint representations of gene expression and morphology. These are being used to improve the prediction of cell type annotations (Bao et al. 2022; Monjo et al. 2022), perform the reconstruction of cell morphology from gene expression data (Lee and Welch 2022), and to infer single-cell transcriptomic profiles from histology staining data (Comiter et al. 2023).

Spatial transcriptomics has also been combined with other microscopy modalities. Its combination with cryo-electron microscopy merges ultrastructure morphology with spatial transcriptomics. In mouse brain preparations, this approach unveiled transcripts enriched in reactive microglia, characterized by a high lysosomal content (Androvic et al. 2023). Information of genes correlated to other subcellular units such as mitochondria, endoplasmic reticulumm, or secreted vesicles could give insight into human diseases. Similarly, its combination with mass spectroscopy imaging (MSI) merges spatial transcriptomics with metabolomics. A caveat of these methodologies is that both techniques are destructive and require using two adjacent tissue slices. However, a new spatial multimodal protocol might allow for the performance of MALDI-MSI and spatial transcriptomics in the same tissue slice (Vicari et al. 2023). Finally, Raman Spectroscopy is also being used to measure the vibrational spectra of histological samples (Chen et al. 2022a). An advantage of Raman Spectroscopy is that it is a non-destructive, label-free approach to measure chemical species in cells (Cao et al. 2016). So far, this approach has been combined with spatial transcriptomics in cell cultures (Kobayashi-Kirschvink et al. 2021). Its extension to tissue slices would enable the sampling of the chemical properties of a tissue—such as its lipid composition—and link them to its transcriptional profile.

## Calcium imaging

Most morphological characteristics discussed earlier represent stable features that change more slowly than the dynamic regulation of gene expression. To adapt to their environment, cells utilize intracellular messengers to signal and orchestrate complex responses. Among these molecules, Ca^2+^ ions play a crucial role, regulating nearly every cellular process, including cell morphology (Clapham 2007). Ca^2+^ signaling can influence both transcriptomic regulation, directing the synthesis of RNA transcripts, and epigentic modifications, ensuring a lasting response (Hernández-Oliveras and Zarain-Herzberg 2024).

**Fig. 2:**
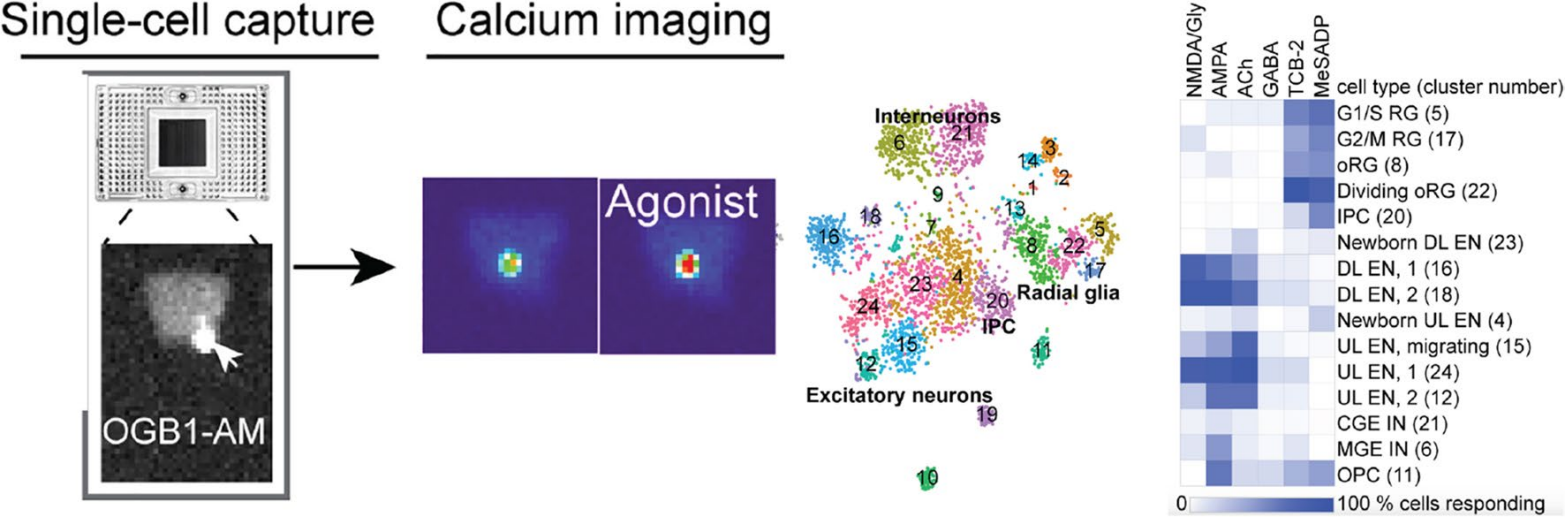
Microfluidics approach to combining Ca2 + imaging with scRNAseq. Individual progenitor cells are captured in individual chambers and treated with neurotransmitter receptor agonists. Isolated cells are then processed to obtain scRNAseq libraries, sequenced, and clustered to identify cell types.  The percentage of cells showing Ca^2+^ responses to each agonist is identified for each cell type. Reproduced from Lock et al. (2015)

Consequently, the concentration of Ca^2+^ ions in a cell’s cytoplasm is tightly regulated (~ 100 nM); and intracellularly, Ca^2+^ is stored in compartments such as the endoplasmic reticulum and mitochondria. Upon stimulation, its concentration rises quickly through the opening of voltage-gated Ca^2+^ channels. This activation initiates signal transduction through multiple molecular pathways (Clapham 2007; Bootman 2012). Fluctuations in intracellular Ca^2+^ levels can be tracked in real-time with fluorescence microscopy. Therefore, measurements of cell activity using high-speed Ca^2+^ imaging and confocal microscopy make it possible to monitor fast responses in cellular and tissue homeostasis. Despite being faster than morphological changes, Ca^2+^ responses are slower than electrophysiological activity (Kulkarni and Miller 2017). However, measuring Ca^2+^ activity does not require specialized patch-clamp equipment, and it is therefore a more accessible approach for most labs. This is further simplified with automated microfluidic devices that capture dissociated cells in individual chambers and perform sequential fluorescence imaging and scRNAseq (Ramalingam et al. 2016). An earlier work used this method to measure functional maturation of progenitor cells during their differentiation into neuronal types (Fig. 2) (Mayer et al. 2019). Limiting factors when using Ca^2+^ imaging to study tissue slices include tissue-penetration depth and the size of the field of view in high-resolution measurements.

### Measurements using calcium dyes

The simplest approach to combine scRNAseq with Ca^2+^ imaging is to use fluorescence microscopy and commercially available Ca^2+^-binding indicators. Most technical aspects discussed in the previous section (Cell Morphology) can be extrapolated to Ca^2+^ imaging by implementing the specific requirements for fluorescence microscopy. A difference with cell morphology is that Ca^2+^ imaging provides access to rapid changes in cell state and activity. Given the large dynamic range of intracellular Ca^2+^ oscillations in cells (milliseconds to minutes), it is important to consider the frequency of Ca^2+^ fluctuations and its relation to the Ca^2+^ indicator kinetics (Smedler and Uhlén 2014). Overall, the selection of an appropriate Ca^2+^ indicator is usually a trade-off between its (i) kinetics, (ii) signal-to-noise ratio, (iii) sensitivity, (iv) cellular or subcellular resolution, and (iv) penetration depth (for in situ measurements) (Grienberger and Konnerth 2012). A popular choice for measurements in the millisecond timescale is Cal-520 due to its signal-to-noise ratio and high temporal resolution (Lock et al. 2015).

### Investigating cell networks

Calcium signaling can propagate as waves through gap junctions across interacting cells. Consequently, another feature of Ca^2+^ imaging is its applicability in studying emergent features such as cell network connectivity (Gosak et al. 2022; Šterk et al. 2023). An illustrative example is insulin secretion by pancreatic β cells. These cells use electrical coupling among themselves and with other cell types through connexin-36 gap junctions. In this way they establish coordinated insulin release in a glucose-dependent manner. Heterogeneity in β cell responses has been well established (Rorsman et al. 2012; Janjuha et al. 2018; Benninger et al. 2014; Kravets et al. 2022; Johnston et al. 2016), and some of this variability is attributed to β cells with higher network connectivity (Johnston et al. 2016). Ca^2+^ recordings revealed that specific β cells initiate Ca^2+^-waves in response to a glucose increase, with other cells following suit (Kravets et al. 2022). Despite this evidence, the molecular characteristics of these cells remain unknown. A combined approach using high-speed confocal Ca^2+^ imaging and scRNAseq has been employed to characterize these cell populations (Chabosseau et al. 2023). To do so, β cells were labeled with a genetically encoded Ca^2+^ reporter (GcaMP6f) and photolabelled based on their connectivity. Consequently, photolabelled cells could be collected using FACS and sequenced (Chabosseau et al. 2023). Although a challenge remains in improving the recovery rate of these ‘photopainted’ cells, this approach demonstrates that Ca^2+^ recordings can be integrated with scRNAseq in closely interacting cells, enabling the measurement of the transcriptome of cells that have been phenotypically characterized based on their network properties.

### Increasing throughput with cell tagging and genetically encoded calcium indicators

Cell tagging based on Ca^2+^ is a promising approach to couple single-cell transcriptomics to cellular activity in vivo on a larger scale. Transcriptional reporter systems, such as the TRAP system, have undergone significant improvements in sensitivity and kinetics (Guenthner et al. 2013). These advancements allow researchers to use these systems for probing the molecular profiles of activated cellular ensembles while simultaneously conducting optogenetic manipulation. For example, scFLARE and FLICRE use a light-gated Ca^2+^ integrator to gain stable genetic access to transiently activated cells (Sanchez et al. 2020; Kim et al. 2020a). To record cell activity, cells are transiently illuminated with blue light for seconds to minutes to uncage the Ca^2+^ indicator (Sanchez et al. 2020). Cells exhibiting Ca^2+^ activity during the illumination window become fluorescently and transcriptionally labeled for FACS and scRNAseq. These methods can bridge functional phenotyping and high throughput scRNAseq.

## Electrophysiology (patch-seq)

**Fig. 3:**
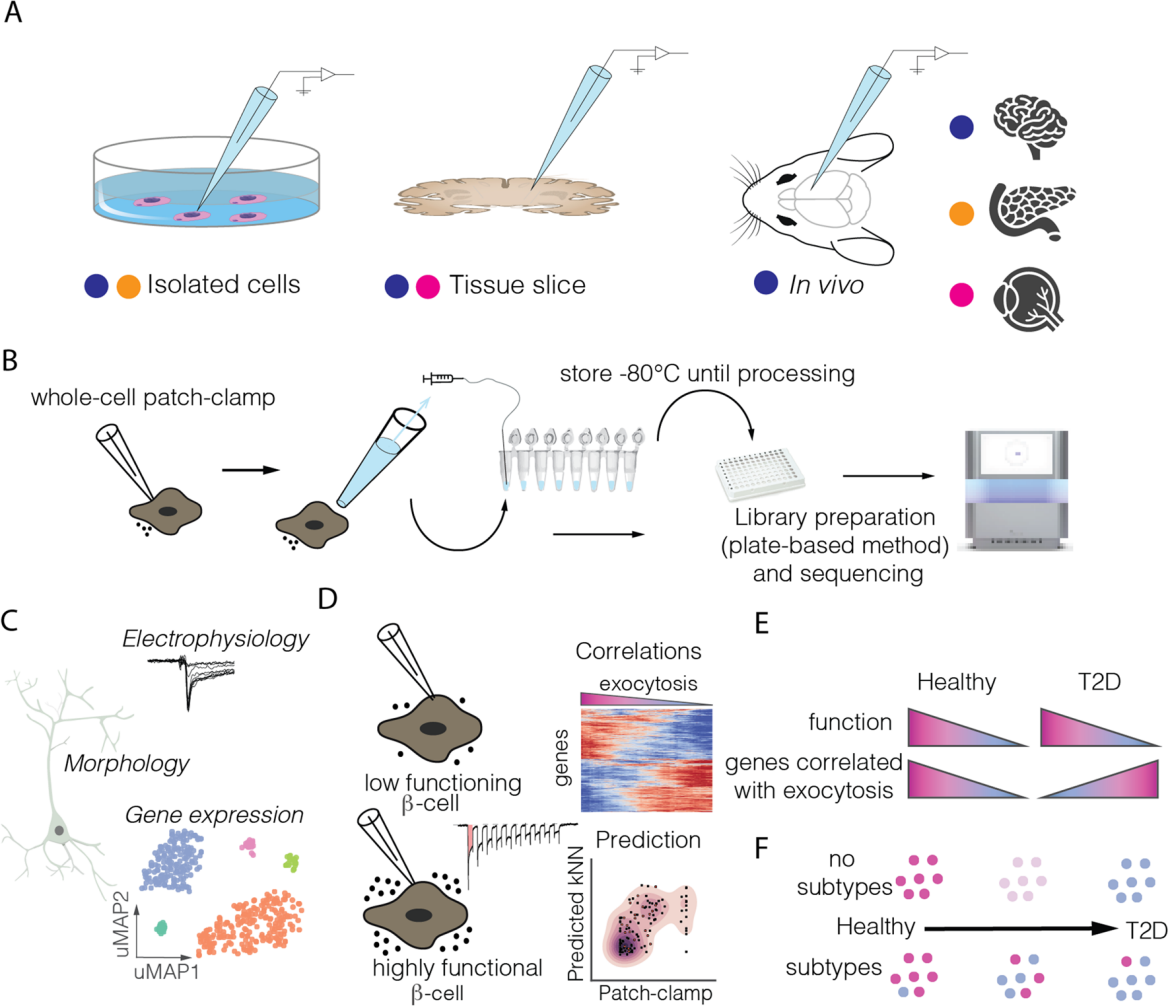
Patch-seq and its applications. **(A**) Patch-seq can be performed in (i) dissociated primary or cultured cells, (ii) acute slices, or (iii) in vivo. Tissues in which each methodology has been applied is shown. **(B**) Overview of experimental patch-seq methodology. After whole-cell patch-clamp electrophysiology, the cell content is aspirated into a microtube with lysis buffer for subsequent library preparation and sequencing. **(C**) In neuronal studies patch-seq can obtain 3 layers of information connected to cell identity: morphology, electrical activity and gene expression. **(D**) In islets patch-seq can be used to infer the secretory capacity of endocrine cells by measuring exocytosis (normalized cell capacitance). **(E**) Patch-seq data in human cells from donors with and without diabetes shows a shift in gene correlations that might be indicative of β cell compensation in T2D. **(F**) Changes in β cells towards a T2D phenotype could take place through a global shift of the entire pool of β cells or through an abundance shift of two distinct β cell subpopulations

Patch-seq was first developed in 2016 (Cadwell et al. 2016; Fuzik et al. 2016; Földy et al. 2016) and represented a tour-de-force in neuroscience. It fuses traditional insights gained from morphological and electrophysiological studies with a comprehensive molecular analysis. These seminal papers focused on interneurons and pyramidal cells in the mouse cortex (Cadwell et al. 2016; Fuzik et al. 2016) and the hippocampus (Földy et al. 2016).They were followed by additional studies on other neuronal subtypes and brain regions by the same labs (Scala et al. 2019; Que et al. 2021; Oláh et al. 2020; Muñoz-Manchado et al. 2018), and others (Ellender et al. 2019; Luo et al. 2019), and studies using isolated iPSC-derived neurons (Bardy et al. 2016; Chen et al. 2016). Patch-seq’s resurgence is linked to the Allen Institute’s brain atlas initiative (Marx 2022), aimed at creating a taxonomy of cell types in the brain (﻿Milo and Phillips 2015; Manno et al. 2018). A detailed review on the application of patch-seq in neuroscience is available in Lipovsek et al. (2021). In addition to neuroscience, the technique has been applied to study cellular heterogeneity in other cell types that regulate their physiology through electrical activity, such as pancreatic islet cells (Kravets and Benninger 2020; Foreman and Wollman 2020; Maltz and Wollman 2022) and retinal cells (Emert et al. 2021) (Fig. 3A). Table 2 summarizes the largest patch-seq studies organized by tissue type.

**Table 2:** Patch-seq datasets with experimental details of each study. Sample size is the number of cells passing QC filters according to the authors (if provided). For studies where we could not find this information we based it on the number of cells available in GEO

Tissue	Species	# Cells	# Donors	Region/Cell types	Assay type	scRNAseq library preparation	Year	Ref	Dataset
Brain	Mouse	58 *(10 *in vivo*)*	-	L1 interneurons and pyramidal cells	Acute-slice & In vivo	Smart-seq2-based protocol	2016	Cadwell et al. 2016)	E-MTAB-4092
Brain	Mouse	83	-	L1-2 CCK + interneurons and pyramidal cells	Acute-slice	Smart-seq2-like protocol (STRT-seq/C1)	2016	Fuzik et al. 2016)	GSE70844
Brain	Mouse	41	-	Hippocampal interneurons and pyramidal cells	Acute-slice	SMARTer Ultra Low Input RNA kit	2016	Földy et al. 2016)	GSE75386
Brain	Human	56	-	iPSC-derived neurons	Dispersed cells	SMARTer Ultra Low Input RNA kit	2016	Bardy et al. 2016)	NA
Brain	Human	20	-	iPSC-derived neurons	Dispersed cells	NEBNext Ultra DNA library Prep Kit	2016	Chen et al. 2016)	GSE77564
Brain	Mouse	98	-	interneurons from striatum	Acute-slice	Smart-seq2-like protocol (STRT-seq/C1)	2018	Muñoz-Manchado et al. 2018)	GSE119248
Brain	Mouse	7	-	GABAergic neurons in hippocampus	Acute-slice	SMART-Seq v4 ultra low input RNA kit	2019	Luo et al. 2019)	GSE109755
Brain	Mouse	110	-	L4-5 neurons from neocortex	Acute-slice	SMART-Seq v4 ultra low input RNA kit	2019	Scala et al. 2019)	GSE134378
Brain	Mouse	370^a^	-	Neural progentior cells in neocortex	Acute-slice	Smart-seq2-based protocol	2019	Ellender et al. 2019)	NA
Brain	Mouse	65	-	apical progenitor cells in neocortex	Acute-slice	SMART-Seq v4 3′ DE Kit	2019	Oberst et al. 2019)	GSE122644
Brain	Mouse	53	-	primary visual cortex	In vivo	Smart-seq2-based protocol	2020	Liu et al. 2020)	GSE115997
Brain	Rat	17	-	CCK + interneurons from hippocampus	Acute-slice	SMART-Seq v4 ultra low input RNA kit	2020	Oláh et al. 2020)	GSE133951
Brain	Mouse	220	-	Cortical neurons	Acute-slice	Smart-seq2-based protocol	2020	Cadwell et al. 2020)	GSE140946
Brain	Mouse	128	-	Parvalbumin-interneurons from hippocampus	Acute-slice	SMART-Seq v4 ultra low input RNA kit	2021	Que et al. 2021)	GSE142546
Brain	Mouse	4,270^b^	-	Cortical GABAergic interneurons	Acute slice	SMART-Seq v4 ultra low input RNA kit	2020	[*101*	Brain-Map^f^
Brain	Mouse	1,237^c^	-	primary motor cortex	Acute slice	Smart-seq2-based protocol	2021	*109*, *111*]	BICCN^g^
Brain	Human	25	11	L5 pyramidal neurons	Acute slice	SMART-Seq v4 ultra low input RNA kit	2021	Kalmbach et al. 2021)	Brain-Map^f^
Brain	Human	385	56	L2-3 pyramidal neurons from neocortex	Acute slice	SMART-Seq v4 ultra low input RNA kit	2021	Berg et al. 2021)	Brain-Map^f^
Pancreas	Human	1,369	34	Endocrine islet cells and other pancreatic cells	Dispersed cells	Smart-seq2-based protocol	2020	Camunas-Soler et al. 2020)	GSE124742
Pancreas	Human	640^d^	19	Endocrine islet cells and other pancreatic cells	Dispersed cells	Smart-seq2-based protocol	2022	Dai et al. 2022)	GSE164875
Pancreas	Human	189	3	Cryopreserved endocrine islet cells	Dispersed cells	Smart-seq2-based protocol	2022	Marquez-Curtis et al. 2022)	PancDB^h^
Pancreas	Mouse	23^e^	-	Pancreatic β cells	Intact islets	NEBNext single-cell/low input RNA kit	2023	Chabosseau et al. 2023)	NA
Retina	Mouse	472	-	Retinal ganglion cells	Retinal whole-mount	Smart-seq2-based protocol	2022	Huang et al. 2022)	GSE137400

^a^For this study, we inferred the number of cells by adding the information provided across figures

^b^Also a number of macaque (Mayr et al. 2019) and human cells (Mayr et al. 2019)

^c^Companion paper contains 133 (mouse), 6 (macaque), 391 (human)

^d^Additional dataset with > 100 mouse cells

^e^This study uses Ca^2+^ imaging and tagging instead of patch-seq

^f^https://portal.brain-map.org/explore/classes/multimodal-characterization

^g^https://www.biccn.org/data

^h^https://hpap.pmacs.upenn.edu/

### Methodological aspects

The main principle behind patch-seq is to aspirate the cellular content (or soma for neurons) of a single patch-clamp-recorded cell and then collect it into a PCR tube containing lysis buffer. This process allows for in-tube reverse transcription and PCR amplification using scRNAseq (Fig. 3B) (Fuzik et al. 2016). In neuroscience, electrophysiological measurements are usually performed in acute slice preparations, although in vivo patch-seq measurements are possible (Cadwell et al. 2016; Liu et al. 2020). In islet research, measurements are commonly performed in dispersed islet cells due to specific challenges in pancreatic tissue slice preparations, the main issues being its high RNAse content and the small size of endocrine α and β cells. In general, it is important to maintain RNase free conditions during sample collection to avoid mRNA degradation. This can be achieved by using RNase inhibitors in the intracellular solution and lysis buffer and by decontaminating lab surfaces. The former may require adjusting the osmolarity of the intracellular solution (Cadwell et al. 2017a; Lipovsek et al. 2020). For in situ measurements, it is possible to add a dye to visualize the pipette tip and surrounding cells, and to fill in the target neuron with biocytin for subsequent immunostaining and morphological reconstruction (Cadwell et al. 2016; Scala et al. 2019; Gouwens et al. 2020). Another common approach is to increase the size of the patching pipette and use reduced volumes of intracellular solution to facilitate the aspiration of the cell content. This also minimizes dilution of the mRNA into the pipette. A critical aspect to obtain high quality mRNA is to aspirate the cell nucleus (Cadwell et al. 2017a; Tripathy et al. 2018). Once the cell is collected, it is dispensed in a lysis buffer tube and processed with standard plate-based scRNAseq protocols such as SmartSeq2 (Fig. 3B) (Picelli et al. 2014).

A key aspect is that index information needs to be preserved to match the electrophysiological and sequencing data. This makes patch-seq incompatible with high-throughput droplet-based methods such as 10X or Drop-seq (Macosko et al. 2015). It is also important to collect positive and negative controls to verify matching between both data modalities and to monitor contamination. The whole protocol is time-consuming, and in optimal conditions, a skilled electrophysiologist can collect 6–7 in situ cells with morphological reconstruction, and up to 40 cells using short recording protocols in cell cultures (Camunas-Soler et al. 2020; Marx 2022). A detailed overview of the patch-seq methodology can be found in previous review articles (Lipovsek et al. 2021), and in book chapters (Dallas et al. 2188). Additionally, step-by-step protocols are available for neurons (Cadwell et al. 2017b), small interneurons (Lipovsek et al. 2020), and cultured human (iPSCs) (Hurk et al. 2018). An end-to-end experimental workflow with benchmarked software and data analysis tools is found in Lee et al. (2021).

### Patch-seq in neuroscience

In neuroscience, patch-seq resolves transcriptome-wide gene expression variation in morphologically defined neurons. This is uncovering cell-type-specific determinants of neuronal cytoarchitecture and can enhance neuronal classification (Fig. 3C) (Fuzik et al. 2016). The Allen Institute and the BRAIN Initiative Cell Census Network (BICCN) are using patch-seq to create a multimodal cell census and Brain Atlas (Table 2) (Gouwens et al. 2020; BRAIN Initiative Cell Census Network (BICCN) 2021; Berg et al. 2021). These large-scale initiatives have succeeded at obtaining patch-seq data for thousands of neurons in the primary motor and visual cortex in rodents (Gouwens et al. 2020; BRAIN BRAIN Initiative Cell Census Network (BICCN) 2021; Bakken et al. 2021) as well as human pyramidal neurons (Berg et al. 2021; Kalmbach et al. 2021). Patch-seq is also used by individual labs. A recent study investigated cortical organization during development in the mammalian cortex. This has revealed that clonally-related neurons are more likely to be connected vertically across layers than within layers (Cadwell et al. 2020). Patch-seq has also been combined with in situ hybridization techniques (FISH) to investigate synaptic connections between excitatory and inhibitory neurons in the human cortex (Kim et al. 2020b). An advantage of patch-seq over standard scRNAseq methods in the brain is its capability to correlate gene expression with neuronal electrophysiological features. This has revealed correlations between transcriptome and neuronal position (Gouwens et al. 2020; Scala et al. 2021), and a transcriptional gradient in striatal interneurons that correlates to fast-spiking patterns (Muñoz-Manchado et al. 2018; Stanley et al. 2020). Overall, patch-seq refines cell-type classifications and validates findings across data modalities in neuroscience.

### Patch-seq in pancreatic islet research

In pancreatic islet research, patch-seq can be used to study the main cell types regulating glucose homeostasis, namely α and β cells (Dai et al. 2022; Camunas-Soler et al. 2020). Functional heterogeneity in islet cells has been long recognized, with variations in β cell insulin release, electrophysiological activity and Ca^2+^ flux (Rorsman et al. 2012; Pipeleers 1992; Salomon and Meda 1986). In parallel to this, single-cell studies found substantial molecular heterogeneity within islet cells, reporting multiple subtypes of β cells (Dorrell et al. 2016; Segerstolpe et al. 2016; Muraro et al. 2016; Baron et al. 2016; Tritschler et al. 2017). It has proved generally challenging to establish connections between both types of measurements (Kravets and Benninger 2020; Wang and Kaestner 2018). Due to the main role of β cells in progression to T2D (Ashcroft and Rorsman 2012) most attention has been devoted to these cells. A distinctive feature of patch-seq in islet research is its ability to measure correlations with exocytotic function (Fig. 3D). Exocytotic function is a hallmark property of β cells that is impaired in T2D. Therefore, measuring correlations between exocytotic function and gene expression helps identify genes involved in T2D progression. In the first patch-seq study in islet-cells (Table 2), new genes that correlate to β cell exocytosis were discovered and used to build predictive models of electrophysiology. By comparing gene correlations between healthy donors and those with T2D, this work identified a transcriptional shift in T2D, hinting at an underlying mechanism of islet compensation under metabolic stress (Fig. 3E) (Camunas-Soler et al. 2020). In parallel, this work also showed that patch-seq can be performed in cryopreserved samples (Camunas-Soler et al. 2020), making it possible to study bio-banked samples (Lyon et al. 2016; Marquez-Curtis et al. 2022). A second patch-seq study focused in α-cells, whose (dys)regulation is also important in diabetes (MacDonald et al. 2007; Girard 2017; Gromada et al. 2018). This work showed that α-cells in T2D show a heterogeneous loss of function, which is linked with the cell maturation state and to dysregulation of P/Q-type Ca^2+^ channels (Dai et al. 2022). Overall, patch-seq data from islet cells is continually being uploaded to the PancDB site as part of the Human Pancreas Analysis Program – T2D (K. H. Kaestner et al. 2019; Shapira et al. 2022).

Patch-seq data from these studies has also been used to map and infer electrophysiological function from standard scRNAseq datasets. Using this approach, co-expression of two islet-specific transcription factors (MAFA/MAFB) has been suggested to be predictive of functional maturation in β cells (Shrestha et al. 2021). The converse also holds true, and hypotheses derived from patch-seq datasets are being investigated in scRNAseq atlases. Recently, a mouse islet atlas has identified a mechanism of insulin secretion and diabetic compensatory response compatible with T2D patch-seq data (Hrovatin et al. 2022). An unresolved question in patch-seq studies has been whether compensatory mechanisms in T2D would affect all or only a subset of β cells (Fig. 3F) (Wang et al. 2023b). The second option could indicate that a subtype of β cells, which is prone to dysfunction and metabolic stress, becomes enriched in T2D. A recent integration of patch-seq data with single-cell multiomics suggests that the latter may be true (Fig. 3F) (Wang et al. 2023b). Overall, patch-seq in islet research can inform candidate gene selection for in-depth mechanistic studies. It can also characterize cellular subtypes with specific functional signatures related to pathophysiology.

### Patch-seq in the retina

The retina is a relatively accessible tissue with a high diversity of cell types that can be defined based on morphology, function, or transcriptional profile (Rheaume et al. 2018). Additionally, it can be studied in flat preparations, making it an ideal tissue for multimodal methods. Recently, mouse retinal ganglion cells (RGCs) have been characterized with patch-seq (Table 2) (Huang et al. 2022). Another unique feature of retinal studies is that the retinal whole-mount preparation can be used to study a neural circuit under external light stimulations (Masland 2012). Patch-seq has been used in this case to characterize the light response of RGCs under spotlight stimuli which improved cell type annotations, and made it possible to identify genes that characterize RGCs that respond either to light increments or decrements (Huang et al. 2022).

## Conclusions and future directions

Single-cell technologies are revolutionizing the way we approach biology and our ability to measure cellular diversity and heterogeneity. Differences in molecular composition, structure, and morphology of cells are a critical aspect of cell identity and are connected to its physiological function. Methods to merge single-cell transcriptomics with other cellular phenotypes such as morphology or electrophysiological activity enable a more complete understanding of cellular heterogeneity and function, improving our ability to classify cell types and states.

Neuroscience has pioneered the development of multimodal profiling to survey the vast diversity of neuronal cell types. Among these methods, patch-seq is a powerful approach due to its ability to merge transcriptome-wide molecular analysis with morphology and electrophysiology. Other fields are following suit, and multimodal integration of cell physiology and transcriptomics is being used in multiple tissues. For instance, patch-seq is becoming a popular tool in pancreatic islet research. A caveat of patch-seq in islet cells is that it has only been performed in dissociated cells, in contrast to in situ and in vivo studies in neuroscience. Improvements in methods for long-term culture of tissue slices and new phenotyping tools should enable in situ measurements in the future (Speier and Rupnik 2003; Marciniak et al. 2014; Huang et al. 2011). The development of soft-semiconductor electronics and microelectrode array systems might enable the recording of tissue-wide electrophysiology (Floch et al. 2022; Li et al. 2021) in parallel to single-cell transcriptomics in multiple tissues. These systems could also be used to quantify the functional development deep inside 3D organoids. Additionally, given that soft microelectronic devices can record the electrical activity of a cell without perforating the cell membrane, the measurement is non-destructive, and the cell properties can be followed over time. This could be combined with cytoplasmatic sampling, which makes it possible to sample the RNA content of the same cell at different time points (Chen et al. 2022b). This approach could be used to simultaneously track morphological and transcriptional dynamics of cell populations during development or under external perturbations.

Currently, the use of approaches that integrate functional phenotyping and single-cell transcriptomics has remained predominantly limited to specialized laboratories, primarily due to the demanding nature of obtaining both measurements from the same cell. However, new methods to increase throughput, such as automation or cellular tagging and barcoding, holds the potential to broaden the accessibility of these technologies across a wider range of researchers in genomics in physiology. Additionally, progress in combining functional phenotyping with spatial transcriptomics will offer new possibilities for a detailed mapping of cell phenotypes in situ and advance our understanding of tissue physiology.

## Limitations of this study

Single-cell genomics is an extremely fast-paced field, and although I have tried to cover the most recent literature it is inevitable that some relevant references might have been unduly omitted. I apologize in advance to these colleagues whose work might have been overlooked.

## Data Availability

No data was generated in this study.

## Code availability

No new code was generated in this study.
